# Functional glycosylation in the human and mammalian uterus

**DOI:** 10.1186/s40738-015-0007-0

**Published:** 2015-10-23

**Authors:** Gary F. Clark

**Affiliations:** Division of Reproductive and Perinatal Research, Department of Obstetrics, Gynecology and Women’s Health, University of Missouri, 1 Hospital Drive HSC M658, Columbia, MO 65211 USA

**Keywords:** Glycosylation, Oligosaccharides, Lectins, Mass spectrometry, Endometrium, Decidua, Placental villi, HELLP, Pregnancy induced hypertension, Preterm birth

## Abstract

**Background:**

Glycosylation is the most common and structurally diverse of all the post-translational modifications of proteins. Lipids and extracellular matrices are also often glycosylated. The mammalian uterus is highly enriched in glycoconjugates that are associated with the apical surfaces of epithelial cells and the secretions released by both epithelial and stromal cells. These glycoconjugates interact primarily with sperm, the implanting embryo, the fetus, and any pathogen that happens to gain entry into the uterus. Secretions of the endometrial glands increase substantially during the luteal phase of the menstrual cycle. These secretions are highly enriched in glycoproteins and mucins that promote specific uterine functions.

**Findings:**

Lectins and antibodies have been employed in the majority of the studies focused on uterine glycosylation have employed to define the expression of carbohydrate sequences. However, while these studies provide insight about potential glycosylation, precise information about glycan structure is lacking. Direct sequencing studies that employ biochemical or mass spectrometric methods are far more definitive, but have rarely been employed with uterine glycoproteins. Both lectin/antibody binding and direct carbohydrate sequencing studies that have been focused on the mammalian uterus are reviewed. The primary functional role of the eutherian uterus is to facilitate fertilization and nurture the developing embryo/fetus. Trophoblasts are the primary cells that mediate the binding of the embryo and placenta to the uterine lining. In mammals that utilize hemochorial placentation, they invade the decidua, the specialized endometrial lining that forms during pregnancy. Trophoblasts have also been analyzed for their lectin/antibody binding as a complement to the analysis of the uterine cells and tissues. They will also be reviewed here.

**Conclusions:**

The functional roles of the glycans linked to uterine and trophoblast glycoconjugates remain enigmatic. Another major question in the human is whether defects in placental or uterine glycosylation play a role in the development the Great Obstetrical Syndromes. More recent findings indicate that changes in glycosylation occur in trophoblasts obtained from patients that develop preeclampsia and preterm birth. The functional significance of these changes remain to be defined. Whether such shifts happen during the development of other types of obstetrical syndromes remains to be determined.

## Introduction

Glycosylation is a specific type of post-translational modification of proteins, lipids and other cellular components that is universally observed throughout the plant and animal kingdoms [1, 2]. The plasma membranes of cells, extracellular matrices and connective tissues are the primary sites where abundant glycosylation is observed [2]. Carbohydrate sequences expressed on the outer surfaces of cells participate in binding to other cell types and crucial signaling events during both physiological and pathological states [3, 4]. It is therefore not that surprising that glycans are profusely expressed in the mammalian uterus, an organ that must undergo many different transformations to support fertilization and subsequent fetal development [5, 6]. Though there is currently rather limited data available about the precise sequences of glycans linked to uterine glycoconjugates, the advent of ultrasensitive mass spectrometric (MS) methods combined with a greater appreciation of the role of glycosylation in reproduction should provide strong incentives in the future for the glycomic analysis of cells and tissues within this organ [7–10]. Here both classical and modern studies focused on uterine glycosylation will be reviewed.

### Glycans as essential functional groups that facilitate reproduction

Monroy provided the first convincing evidence that carbohydrate recognition is essential for sperm-egg binding. Specifically, he proposed that polysaccharides or glycans presented on the egg jelly coat of marine organisms were recognized by lectin-like egg binding proteins on the surface of sperm, enabling robust gamete binding [11]. This model for specific cell-cell recognition relies on the strict regulation of carbohydrate expression on both sperm and eggs. The carbohydrate ligands for sperm binding must be expressed at elevated levels on the extracellular matrix of the egg, but not on the sperm surface where they could interact with the lectin-like egg binding proteins on the plasma membrane and inhibit binding. This same type of regulation also applies to cell signaling events involving the specific recognition of carbohydrate ligands. Expression of the carbohydrate ligand for a receptor on the same surface as the receptor could inhibit signaling.

This logic can be directly applied to initial murine and human sperm-egg binding, where substantial evidence supports a specific carbohydrate binding specificity. Data obtained from many different studies indicate that the major egg binding protein on mouse sperm interacts with triantennary and tetraantennary N-glycans terminated with β-linked Gal presented on the constituent glycoproteins that form the zona pellucida (ZP) [12–14]. Similarly, the major egg binding protein on sperm has been proposed to recognize multivalent sialyl-Lewis^x^ sequences presented on both N- and O-glycans of ZP glycoproteins during initial gamete binding in humans. Clearly, these carbohydrate ligands for murine and human gamete binding should not be expressed on the plasma membranes of mouse and human sperm. Otherwise, they could interact with the lectin-like egg binding proteins to block binding. Glycomic studies have demonstrated this restriction in the human model. As noted previously, sialyl-Lewis^x^ (sLe^x^) is the carbohydrate ligand on the ZP that mediates human sperm-ZP binding [15]. However, glycomic analysis did not indicate the expression of sLe^x^ on human sperm glycans, in spite of the presence of both highly sialylated and fucosylated glycans [16]. Evidence supporting this type of restriction in other species can be obtained in the future by performing detailed glycomic analyses of mature sperm and ZP glycoproteins.

It is quite easy to understand how receptor-ligand systems involving protein-protein interactions between different cell types could be regulated by the genome, but much more difficult for carbohydrate-dependent interactions [17]. Unlike proteins, carbohydrate sequences are assembled via a template-independent process [2]. Their synthesis relies upon the availability of specific modification enzymes (glycosyltransferases, glycosidases), sugar nucleotide sugars and protein substrates [2, 18, 19]. Other relevant factors include the competition for substrate glycans by different enzymes, and the organization of enzymes into complexes or organelles like the Golgi apparatus [20, 21].

Precisely how the expression of complex glycans is regulated remains an enigma. Nonetheless, recent evidence indicates that glycans have been employed as functional groups since the initiation of life on this planet. The synthesis of carbohydrate sequences has been documented in Archaean prokaryotes that date back more than 3500 mya [22]. Recent findings suggest that glycosylation was likely essential to enable these ancient organisms to survive the very harsh environmental conditions that existed during the early stages of earth’s history. The functional roles for glycans has greatly expanded over the eons. Evidence for sexual reproduction has been identified in the fossils of bangiacean red algae (*Bangiomorpha pubescens*) that date back 1200 mya [23]. These results indicate that the pathways for regulating the expression of carbohydrate functional groups and their cognate receptors on different gametes have likely existed for millennia. The fact that they are still employed in humans confirms that the functional roles requiring carbohydrate recognition remain under positive selection.

Abnormal glycosylation contributes to the development of many different pathological states in humans [2]. The Great Obstetrical Syndromes (preterm labor, preeclampsia, intrauterine growth restriction, preterm premature rupture of membranes, late spontaneous abortion, abruptio placentae) remain the issues of foremost concern for clinicians devoted to the delivery of healthy infants [24–28]. All of these pathological states are clearly associated with disorders of deep placentation [29]. Whether any of these syndromes are the result of defective uterine glycosylation has yet to be determined. Unlike genetic or epigenetic changes, subtle shifts in glycosylation are completely invisible to the current methods of genomic analysis. However, they can be readily revealed by careful glycomic analysis of glycoconjugates and whole cell types isolated from normal and pathological tissue samples [7, 30, 31]. The great majority of the studies focused on analyzing uterine glycosylation have been performed with lectins and carbohydrate-specific antibodies. However, ultrasensitive MS analyses will be essential to precisely define discrete differences in glycosylation between normal and pathological states in the human uterus that could result in the development of these obstetrical syndromes [7–10]. Studies focused on the expression of glycosyltransferase genes in the uterus will also be reviewed.

### Analysis of human uterine glycoconjugates with carbohydrate binding proteins

The human uterus is highly enriched in glycoconjugates that are associated with the apical surfaces of epithelial cells and the aqueous secretions released by both epithelial and stromal cells. These glycoconjugates interact primarily with sperm, the implanting embryo, the fetus, and any pathogen that gains entry into the uterus. Secretions of the endometrial glands increase substantially during the luteal phase of the menstrual cycle. These secretions are highly enriched with growth factors and nutrients that support the implantation of the embryo and its subsequent development into a viable fetus.

Glycosylation in the human uterus has been studied primarily by employing lectins and carbohydrate-specific antibodies [32, 33]. This approach was initially necessary because of the limited amount of available tissue/cells and the relative insensitivity of the methods of carbohydrate structural analysis. Lectins are proteins that recognize and bind to carbohydrate sequences that express specific structural features [34]. Many lectins with different carbohydrate binding specificities have been isolated and purified to homogeneity since 1970 [34–38]. The major lectins employed to profile glycosylation in the many different human cell types are shown in Table 1.

**Table 1:** Binding specificities of lectins commonly employed to analyze glycosylation

Abbreviation	Source	Carbohydrate Binding
AAA	*Anguilla anguilla*	Fucosylated type 1 chains (H1, Lewis^a/b^)
ALA	*Aleuria aurantia*	Fucose linked α1-6 to N- acetylchitobiose core of N-glycans
BPA	*Bauhinia purpurea*	Galβ1-3GalNAc, α-linked GalNAc
Con A	*Concanavalia ensiformis*	Terminal α-linked mannose; high mannose and biantennary type N-glycans
DBA	*Dolichos biflorus*	A blood group antigen Terminal α/β-linked GalNAc
DSA	*Datura stramonium*	N-acetyllactosamine, Linear polylactosaminoglycans
ECA	*Erythrina cristagalli*	Galβ1-4GlcNAc (lacNAc)
E-PHA	*Phaseolus vulgaris*	Biantennary/triantennary bisecting type N-glycans
GNA	*Galanthus nivalis*	High mannose type N-glycans primarily via terminal Manα1-3Man
GS-I	*Griffonia simplicifolia*	Galα1-3/4Gal, GalNAcα1-3Gal
GS-II	*Griffonia simplicifolia*	Terminal α/β-linked GlcNAc
LBA	*Phaseolus lunatus*	A blood group (GalNAcα1-3[Fucα1-2]Gal)
LCA	*Lens culinaris*	N-glycans with fucose linked α1-6 to the N-acetylchitobiose core
LEA	*Lycopersicon esculentum*	Polylactosamine sequences
LTA	*Tetranogolobus purpureus*	H2 antigen, Lewis^x^, Lewis^y^
L-PHA	*Phaseolus vulgaris*	β-1-6 linked lacNAc in tri-/tetraantennary N-glycans
MAL-II	*Macckia amurensis*	NeuAcα2-3Galβ1-4GlcNAc
MPA	*Maclura pomifera*	Tn antigen (α-linked GalNAc) or T antigen (Galβ1-3GalNAc)
PNA	*Arachis hypogaea*	Galβ1-3GalNAc
PSA	*Pisum sativum*	N-glycans bearing fucose linked α1-6 to the N-acetylchitobiose core
PWM/PAA	*Phytolacca americana*	Branched polylactosaminoglycans
RCA-I	*Ricinus communis*	Galβ1-4GlcNAc > Galβ1-3GlcNAc
RCA-II	*Ricinus communis*	Terminal β-linked Gal or GalNAc
SBA	*Glycine max*	α-linked GalNAc > α-linked Gal
SJA	*Sophora japonica*	α/β-linked GalNAc > α/b-linked Gal
SNA	*Sambucus nigra*	NeuAcα2-3Galβ1-4GlcNAc
STA	*Solanum tuberosum*	Polylactosamine sequences
UEA-1	*Ulex europaeus-1*	H2 antigen (Fucα1-2Galβ1-4GlcNAc) Lewis^y^
VVA	*Vicia villosa*	GalNAcα1-Ser/Thr and GalNAcα1-3Galβ1-
WFA	*Wisteria floribunda*	GalNAcα1-6Galβ1-, GalNAcα1-3Galβ1-
WGA	*Triticum vulgaris*	Multivalent terminal NeuAc, polylactosamine sequences
sWGA	Succinylated WGA	Polylactosamine sequences

Though lectins are useful tools, they cannot provide precise details about glycan expression, due to their potential for cross reactivity with unknown carbohydrate sequences and the enormous structural diversity of glycans, especially those that possess multiple non-reducing terminals due to branching. However, when employed in conjunction with ultrasensitive MS sequencing tools, lectins can be very useful for precisely defining structure-function relationships. The unambiguous identification of the glycoprotein ligands for DC-SIGN in human seminal plasma could only be accomplished by employing lectin affinity chromatography in conjunction with glycomic and proteomic analyses, as demonstrated in a recent study [39].

Differential agglutination of human tumor cells compared to normal progenitor cells by wheat germ agglutinin (WGA) was initially reported in 1965 [40]. This observation led many investigators working in cancer research to employ lectins in their comparative studies of normal versus tumorigenic tissues and cell types. This interest in differential glycosylation during tumorigenesis also stimulated many investigators to define the carbohydrate binding properties of the lectins shown in Table 1.

Many different lectin binding studies have been performed on tissue samples obtained from the human uterus and cervix (Table 2). In an early study, Rowinski and coworkers reported that fibroblasts obtained from the normal human cervix were not agglutinated by the lectin Concanavalin A (ConA). By contrast, fibroblasts underlying different cervical cancer lesions became agglutinated with lower concentrations of this lectin as the tumor progressed [41]. Kluskens et al. [42] analyzed the binding of 7 FITC-labeled lectins to proliferative, hyperplastic and cancerous endometrial samples. They were able to define differences in the binding of WGA and ConA to these samples. The sialic acid binding lectin from *Limulus polyphemus* was employed to investigate the changes in the expression of sialylated glycoconjugates in human endometrial adenocarcinoma after treatment with medroxyprogesterone acetate [43]. They observed specific quantitative and qualitative differences in lectin binding after therapy with this hormone.

**Table 2:** Lectin binding to human uterine tissues

Tissue/cell type^a^	Lec tins Employed	Cancer status^b^	Ref.
U, C	ConA	N, M	42
E	*Limulus polyphemus*	M	43
U, C	Panel of 13 lectins	N	44
E	PNA, UEA, WGA, ConA	N	45
C	WGA, PNA	N, M	46
U, C	Panel of 19 lectins	N	47
E	UEA-1, GS-I, DBA	N, M	48
C	Panel of 11 lectins	N, M	49
C	Panel of 12 lectins	N, M	50
C	UEA, DBA, ConA, PHA	N, M	51
O	PNA, SBA, DBA, WGA, ConA, LTA, UEA	N	52
U	PNA, ECA	N	53
C	PWM, WGA	N, M	54

^a^Abbreviations: *U* uterus, *C* cervix, *E* endometrium, *O* oviduct

^b^N Normal, *M*, Malignant

Damjanov and coworkers investigated the binding of a panel of 13 different fluoresceinated lectins to normal human endocervical and uterine epithelium at different stages of the menstrual cycle [44]. They reported that MPA, UEA-I, SBA and VVA were selectively bound to the endocervix but not the endometrium, indicating that lectins could be employed to distinguish between epithelia at different uterine sites. They also demonstrated that these variations were independent of the menstrual cycle and blood group status. Bychkov and Toto employed the avidin-biotin-peroxidase method to analyze the binding of PNA, UEA-1, WGA and ConA to samples of endometrium during different stages of the menstrual cycle and early pregnancy [45]. They reported very strong binding of PNA and UEA-1 to apical cells during early pregnancy, but only weak binding during the proliferative and secretory phases. WGA and RCA-1 displayed marginal binding to glandular epithelium during the proliferative phase that increased substantially during the secretory phase. This same investigative group employed WGA and PNA as probes to analyze normal, dysplastic and neoplastic cervical epithelium [46]. They observed minimal binding of these lectins to normal squamous epithelium which increased substantially as the lesions became more malignant.

Wan and coworkers used a panel of nineteen FITC-labeled lectins to define the glycosylation of the epithelial surfaces in the human female reproductive tract including the uterus and cervix. They concluded that the distribution of galactosyl residues displayed variations among the organs, unlike mannosylated and fucosylated residues that were more evenly expressed [47]. Tang studied the binding of UEA-1, GS-I (isolectin B4), and DBA to normal and malignant cells of the uterine endometrium [48]. DBA binding decreased while UEA-1 binding increased on tumorigenic luminal cells compared to normal progenitor cells. Griffin and Wells employed a panel of eleven biotinylated lectins to compare the glycosylation of normal cervical glands for comparison with cervical glandular intraepithelial neoplasia and invasive adenocarcinoma [49]. Foster and coworkers analyzed the binding of an extensive panel of lectins to define changes in glycan expression that accompanied the transition from normal to cancerous lesions in human cervical epithelium [50]. They suggested that the expression of novel carbohydrate sequences by cancer cells could substantially promote their invasion and dissemination.

Nagai and associates investigated the binding of UEA, DBA, ConA and PHA to normal and neoplastic glandular epithelium from the human endocervix and endometrium [51]. They reported that the intensity and staining pattern of lectin binding were useful for differentiating between endocervical and endometrial epithelium derived from either normal or neoplastic tissue. The effect of hormonal cycling on the glycosylation of the human oviduct has been studied by employing five horseradish peroxidase-labeled lectins (PNA, SBA, DBA, WGA, ConA, LTA, UEA-I) [52]. Substantial losses in the binding of DBA, WGA and ConA lectins was observed in postmenopausal women compared to the menstruating women. Argueso et al. employed PNA and ECA to analyze the expression of T antigen and N-acetyllactosamine in the human mucin MUC5B during the menstrual cycle [53]. This investigative group reported that the expression of these sequences increased steadily up to midcycle and then dramatically declined by the end of the cycle.

PWM and WGA have recently been employed to analyze the expression of glycans in the human uterine cervix and cervical lesions [54]. Enhanced binding of PWM was observed in squamous carcinoma compared to premalignant lesions (premalignant cervical intraepithelial neoplasia grades 1–3) and normal cervical epithelium. By contrast, the binding of WGA uniformly decreased as the cancerous lesions became more aggressive. Analysis of cervical lesions by lectin blot and enzyme-linked lectin assay (ELLA) also indicated decreased sialylation and fucosylation of cancerous cervical lesions compared to normal epithelium [55].

### Analysis of mammalian uterine glycosylation with carbohydrate binding proteins

The interaction of lectins with uterine tissues has also been studied in many other non-human species (Table 3). Roberts and coworkers isolated a plasma membrane fraction from the luminal surface of the pig uterus during the estrous cycle and early pregnancy [56]. These investigators separated the membrane glycoproteins in this fraction by 2-D gel electrophoresis and stained them with radioiodinated ConA and RCA-I. However, no major changes in either protein expression or glycosylation was detected during either the estrous cycle or early pregnancy with these specific lectin probes [56].

**Table 3:** Lectin and antibody binding to mammalian uterine tissues

Tissue type^a^	Lectins/Antibodies	Animal (species)	Ref.
U	ConA, RCA-I	Pig (*Sus scrofa*)	56
U, O	Panel of 20 lectins	Mouse (*Mus musculus*)	57
U, O	Panel of 11 lectins, 2 antibodies	Monkey (*Cebus apella*)	58
U, P	Panel of 24 lectins	Wallaby (*Macropus eugenii*)	59
E	Panel of 14 lectins	Cat (*Felis catus*)	60
E	Panel of 20 lectins	Rat (*Rattus norvegicus*)	61
E	UEA-1, HPA, WGA	Dog (*Canis familiaris*)	62
E	HPA, WGA, UEA-I, SBA, PNA, LCA	Dog (*Canis familiaris*)	63
U, P	PNA, MPL, WGA, DBA, SBA, RCA-I	Dog (*Canis familiaris*)	64
E	PNA	Dog (*Canis familiaris*)	65
U	GS-I	Mouse (*Mus musculus*)	66

^a^Abbreviations: *U* uterus, *C* cervix, *E* endometrium, *O* oviduct, *P* placenta

In 1983, Damjanov and coworkers employed a panel of fluorescein-conjugated lectins to probe the luminal epithelium lining the murine oviduct and uterus [57]. They were able to demonstrate that WGA, BPA, RCA-I, MPA and UEA-1 displayed differential binding to the epithelial surface of the pregnant versus non-pregnant uterus. This differential binding was also regionally specific with WGA, indicating that changes in the binding of RCA-I, MPA and WGA delineated pregnancy-related changes in the distal oviduct and colliculus tubaris. WGA could also distinguish pregnancy related changes in the proximal oviduct. UEA-I alone reacted exclusively with the epithelium of the non-pregnant uterus. RCA-II reacted preferentially with the epithelium of the oviduct and uterus as compared with its weak reactivity with the stroma. Two lectins (PSA, LCA) reacted selectively with stromal cells of the uterus and oviduct [57].

Aplin and coworkers analyzed the expression of eleven different lectins and two monoclonal antibodies directed against carbohydrate sequences (keratan sulfate, sialyl-Tn antigen) to investigate glycan expression in the oviduct and the endometrium during the luteal phase of *Cebus apella*, a New World monkey [58]. Jones et al. recently employed a panel of twenty-four different lectins to investigate the glycosylation of the placenta and the uterus in a marsupial, the tammar wallaby *Macropus eugenii* [59]. Feline decidual cells displayed weak binding for GS-I, ConA, DBA, DSA, PNA, RCA-I, SBA and SJA in another study [60]. ConA, LCA, SNA, RCA-I, PNA, SBA and HPA were among the lectins that were shown to bind to normal rat endometrium [61]. Several groups have analyzed the binding of a panel of lectins to canine endometrial mucosa that in some cases displayed variation depending on the stage of presentation (immature, oestrus, young anoestrus, aged anoestrus) and disease status [62–65]. Georgiades and coworkers analyzed the binding of GS-I to mouse decidual stromal cells during pregnancy. They reported staining of these cells with GS-I in the venous sinusoid area of the decidual basalis by embryonic day 7.5 of pregnancy and in the entire basalis by embryonic day 10.5 and afterwards [66].

### Biochemical and MS analyses of uterine glycoconjugates in the human and mouse

Though lectins are useful for analyzing the glycosylation of uterine surfaces, they are not nearly as powerful as carbohydrate sequencing tools involving biochemical and MS approaches. Yurewicz and Moghissi isolated sixteen different O-glycans from a pool of human midcycle cervical mucin samples [67]. These investigators subsequently labeled these oligosaccharides with tritium at their reducing ends, enabling them to be sensitively detected during the procedures that enabled their sequence to be defined. Analysis of the neutral O-glycans yielded evidence for the existence of core 2 type O-glycans terminated with H type 2, Lewis^x/a^, and potential Lewis^y/b^ sequences [68]. A core 2 O-glycan terminated with either sialyl Lewis^x^ and/or sialyl Lewis^a^ was the most unusual oligosaccharide revealed during the sequencing of the sialylated fraction [69]. A detailed glycomic analysis of human cervical mucins expressed during the menstrual cycle was more recently performed by employing ultrasensitive MS methods [70]. At least 50 different neutral, sialylated and sulfated O-glycans were detected. The previous findings reported by Yurewicz and Moghissi were confirmed during this study [68, 69]. Hansson and coworkers did not detect any changes in protein or mucin composition in the cervical plug during the menstrual cycle, but they did observe a relative increase in the expression of neutral fucosylated O-glycans during the ovulatory phase [70].

MS methods have also been employed to analyze the glycans associated with specific uterine or decidual glycoproteins. Perhaps the best studied is glycodelin, a glycoprotein of endometrial and decidual origin that was originally isolated by Bohn from the placenta and designated PP14 [71]. The amniotic fluid specific form of glycodelin (GdA) was originally shown to display several different immunomodulatory activities and the ability to block human sperm-ZP binding in the hemizona assay system at low concentrations [72–75]. Glycomic analysis revealed the presence of some very unusual carbohydrate antennae on its N-glycans, such as the fucosylated lacdiNAc sequence (GalNAcβ1-4[Fucα1-3]GlcNAc) [75, 76]. Currently, there are several isoforms of glycodelin that have been identified in the follicular fluid (GdF), cumulus matrix (GdC) and seminal plasma (GdS). Each form has its own specific glycosylation state and biological activities, though the protein component remains unchanged [76].

Carson and coworkers demonstrated that β-estradiol stimulated the incorporation of [^3^H]mannose into mouse uterine glycoproteins by 3–6-fold without stimulating protein synthesis [77]. This increased incorporation was due to enhanced secretion of specific glycoproteins rather than changes in the glycan biosynthetic pathways. This same group later demonstrated that polylactosaminoglycans represent a major fraction of the total glycoconjugates synthesized by epithelial cells but not stromal cells in the mouse uterus [78]. These glycans play a role in cell adhesion processes involving this cell population. The synthesis of these glycans was specifically stimulated by estrogen [79].

Insightful MS analyses have been performed on mouse uterine luminal fluid (ULF) glycoproteins. Glycomic analysis of lipocalin 2 (Lcn2, 24p3) revealed the presence of multiple Lewis^x^ and Lewis^y^ antenna on complex type N-glycans at its only glycosylation site [80]. Lewis^x^, Lewis^y^ and terminal NeuAcα2-6Gal sequences were predominant in mouse ULF glycome. Several other glycoproteins carriers of these antenna were detected in ULF, including abundant lactotransferrin. The exact physiological significance of this unusual glycosylation pattern remains to be determined.

### Analysis of glycosyltransferase gene expression in the human and mammalian uterus

Several groups have studied the expression of different glycosyltransferase genes in the human and mammalian uterus. Levesque et al. confirmed the expression of a UDP-glucuronyltransferase gene in the human uterus by employing an amplification method involving the reverse transcriptase-polymerase chain reaction (RT-PCR) [81]. Kubushiro et al. demonstrated that the level of β1-4 galactosyltransferase enzyme was highly elevated in human endometrial cancer compared to normal endometrium by employing both immunohistochemical approaches and measurement of mRNA levels [82].

Lowe and coworkers employed a similar approach to detect an α1-2 fucosyltransferase (FUT1) in the mouse uterus [83]. Tabak and coworkers confirmed the expression of a UDP-GalNAc:polypeptide N-acetylgalactosaminyltransferase gene that plays a role in O-glycan synthesis in the rat uterus [84]. Robertson and coworkers employed quantitative real-time PCR (qPCR) in murine uterine epithelial cells to demonstrate that α1-2 fucosyltransferase (FUT2) expression in mouse uterine epithelial cells is regulated by leukemia inhibitory factor (LIF) and interleukin-1B (IL-1B) secreted by macrophages [85].

Hormonal regulation of glycosyltransferase gene expression in the uterus has also been demonstrated. Domino and Hurd employed LacZ expression in α1-2 fucosyltransferase (FUT2)-LacZ mice to demonstrate estradiol-regulated endocervical glandular expression during the estrous cycle, hormone replacement and pregnancy [86]. Uchiyama and coworkers analyzed the expression pattern of mRNAs for three hyaluronan synthases (HAS-1, −2, and −3) in the uterine cervix of gravid mice. The expression of HAS-1 and HAS-2 was inhibited by progesterone treatment whereas HAS-3 was substantially increased [87]. Chu and coworkers showed that the progesterone regulated expression of the gene for a specific β1-4 N-acetylgalactosaminyltransferase (B4GALNT2) was required from the time of embryo implantation in mice [88]. Administration of tamoxifen (an estrogen receptor α antagonist) to pregnant mice on d15 resulted in reduce hyaluronan synthase 2 (HAS-2) gene expression in the cervix as quantified by qPCR. This finding also correlated with an overall 50 % decrease in hyaluronan content, indicating that the expression of this glycosaminoglycan is estrogen regulated [89].

### Glycosylation and the implantation of human and mammalian embryos

The mammalian embryo must successfully implant and initiate placentation to obtain nutrients and establish gas exchange. Successful implantation requires a receptive uterine lining and the development of the embryo to the blastocyst stage. The trophectodermal cells of the blastocyst must bind to the luminal epithelial cells of the uterus for implantation to proceed.

Initial implantation of the human embryo into the uterine epithelium has also been proposed to depend on carbohydrate recognition. Hey and Aplin initially reported that sialyl-Lewis^x^ and sialyl-Lewis^a^ are expressed on MUC-1 in the human endometrium [90]. Fisher and coworkers demonstrated that human trophoblasts express L-selectin, an adhesion molecule that is also employed during lymphocyte extravasation from the vasculature [91]. They further reported that human uterine epithelial cells increase their expression of selectin ligands (especially 6-sulfo-sialyl Le^x^) during the temporal window of receptivity. This adhesive interaction is functional, since human trophoblasts specifically bind to beads coated with 6-sulfo-sialyl Le^x^ but not to unrelated carbohydrate ligands [91]. Carson and coworkers subsequently reported that MUC1 also binds to antibodies specific for 6-sulfo-sialyl Lewis^x^ (HECA-452, MECA-79), suggesting a possible role for this mucin in blastocyst implantation [92]. However, while useful, such studies cannot provide precise information about the degree of substitution with these carbohydrate sequences or potential changes in presentation that occur during the receptive period of blastocyst implantation.

Many different cell adhesion molecules have been associated with the implantation of the mouse embryo [33]. There is compelling evidence that lectin-like interactions also play a role in this process. Lundblad and coworkers initially demonstrated that a specific milk oligosaccharide designated lacto-N-fucopentaose I (LNF-1), but not other closely related oligosaccharides inhibited the implantation of mouse embryos by 53 % at a millimolar concentration [93]. This finding was correlated with the expression of LNF-1 on the surface of the murine uterine endometrial epithelium during pregnancy [94]. Another terminal sequence that has been implicated in binding is the H type 1 antigen [93]. Its synthesis relies on the expression of a specific estrogen dependent α1-2 fucosyltransferase [95]. The mRNA for this enzyme is elevated in the preimplantation phase but decreases as implantation proceeds. Intrauterine injection of monoclonal antibodies directed against the Lewis^y^ antigen also inhibits implantation, but only if they are introduced just before this process normally occurs [96]. As noted previously, results obtained in a more recent study have confirmed that Lewis^y^ sequences are also profusely expressed on murine uterine luminal fluid glycoproteins [80].

The isolation and characterization of galectins with different carbohydrate binding specificities led to the concept that there is a glycocode that directs appropriate functions under different physiological states in complex organisms [97, 98]. Subsequent experiments support the hypothesis that there is the specific recognition of a glycocode that functions during the implantation of the mammalian embryo. Jones and Aplin noted that each mammalian species has its own unique pattern of glycosylation of the maternal uterine epithelium based on lectin binding, which they designated as a glycotype [5]. These investigators pointed out that the uterine glycotypes are very similar in cases where interspecies mating results in successful implantation and the development of viable hybrids (horse and donkey, llama and guanaco). They suggest that hybrid embryos are able to implant and develop based partially on their recognition of the shared glycans on the uterine epithelium, i.e., the glycocode [5].

### Analysis of human trophoblast glycosylation

Though not actually a part of the uterus, the placenta functions to provide vital nutrients and gas exchange to support the developing eutherian during pregnancy. The placental cells that make intimate contact with the uterine lining are known as trophoblasts. In addition to providing this vital support, these cells are also crucial for establishing an immunological barrier that protects the histoincompatible fetus from the maternal immune response. Because of their importance and relationships to pathological pregnancy states in humans, trophoblasts have been subjected to many different types of biochemical analyses, including lectin binding studies.

Perhaps the most extensive lectin binding analysis of human trophoblasts obtained from term placenta was carried out by Jones and her colleagues [99]. The results of this study are summarized in Table 4. Ezaki and coworkers also analyzed the binding of a panel of lectins to human trophoblasts [100]. However, these investigators reported much lower binding of ConA, E-PHA and LCA to trophoblasts compared to the findings reported by Jones and coworkers.

**Table 4:** Lectin staining of human trophoblast Domains^ab^

Lectin^c^	Microvillous membrane/ apical cytoplasm	Trophoblast/ basal cytoplasm	Basal plasma membrane/lamina
ConA	4	3	3
PSA	4	3	3
E-PHA	4	3	3
L-PHA	2	0	0
ALA	4	2–3	3
DBA	0	0	0
MPA	4	2	2
DSA	4	3	3
STA	4	2–4	2–4
LEA	1–2	0	2–3
HPA	1	0	0
AHA	0–1	0	0
AHA + N	4	1	1–2
ECA	0–1	0	0
ECA + N	4	2	2–3
SBA	0	0	0
SBA + N	1	0	0
WFA	0	0	0
SNA	0–1	0	0
MAA	4	1	1–2
PAA	0–1	0	1–2
PAA + N	2	0	3
WGA	4	2–3	2–4
WGA + N	1–2	0	1–2

^a^Staining: 0 = negative; 1 = week; 2 = moderate; 3 = strong; 4 = intense

^b^This table was adapted from data reported in [90]

^c^+ N indicates digestion with neuraminidase before lectin staining

At this time, the assignment of any specific functional roles for human trophoblast glycans is purely speculative because of the lack of hard evidence. However, one potential role that such glycans could play involves immune recognition. Human syncytiotrophoblast (STB) come into direct physical contact with uterine NK cells during the early stage of implantation. NK cells are specifically sequestered to the implantation site by MIP-1α, a cytokine secreted by STB [101]. STB on the surface of the placental villi also come into direct contact with immune effector cells in the maternal blood. STB lack human leukocyte antigens (HLA), thus likely avoiding alloimmune responses [102, 103]. However, they encounter circulating maternal natural killer (NK) cells at the villous interface. The lack of HLA class I proteins could potentially put them at risk for cytolysis, but only if they express the appropriate NK cell activating ligands [104, 105].

Of even greater potential immune consequence is the expression of paternal HLA-C by extravillous cytotrophoblasts that invade the decidua and the myometrium [102, 103, 106]. How a powerful histocompatibility-based response directed against this type of trophoblast is avoided during pregnancy is currently unknown. In addition, no studies have demonstrated that differential glycosylation of HLA class I molecules could lead to the evasion of this type of immune response. Clearly, ultrasensitive MS analysis of trophoblast populations in the human placenta combined with other functional studies will be useful for determining if glycosylation plays any functional role in the immune deviations that occur during these interactions.

### Analysis of mammalian trophoblast glycosylation

Trophoblasts and trophectodermal cells from many other species have also been subjected to staining with lectins to analyze glycan expression and detect differences between species. Jones and coworkers performed lectin binding analysis of trophoblasts isolated from humans and four other species that employ hemochorial implantation (lesser hedgehog tenrec (*Echinops telfairi*), spotted hyena (*Crocuta crocuta*), nine-banded armadillo (*Dasypus novemcinctus*), and guinea pig (*Cavia porcellus*)) [99]. These investigators noted that the glycosylation patterns were similar to each other and to human trophoblasts, with only minor differences. They suggested that these findings were evidence for convergent evolution [99]. Lectin binding studies have also been performed on trophoblasts from many other mammalian species. These studies are presented in tabular form in Table 5. The intraepithelial binucleate cells present in ruminants have often been the focus of many of these other investigations.

**Table 5:** Analysis of lectin binding to mammalian trophoblasts

Common Name	Species	Reference
African elephant	*Loxodonta africana*	[114]
Alpaca	*Lama pacos*	[115]
Bovine	*Bos Taurus*	[116]
Cama	(camel/alpaca hybrid)	[115]
Camel	*Camelus dromedaries*	[115]
Chimpanzee	*Pan troglodytes*	[117]
Chinese water deer	*Hydropotes inermis inermis*	[118]
Collared peccary	*Tayassu tajacu*	[119]
Domestic cat	*Felis catus*	[60]
Domestic goat	*Capra aegagrus hircus*	[118]
Domestic pig	*Sus scrofa*	[119]
Egyptian slit-faced bat	*Nycteris thebaica*	[120]
Fallow deer	*Dama dama*	[118]
Giraffe	*Giraffa camelopardalis*	[121]
Greater malayan chevrotain	*Tragulus napu*	[118]
Guinea pig	*Cavis porcellus*	[90]
Hottentot golden mole	*Amblysomus hottentotus*	[122]
Horse	*Equus ferus caballus*	[123, 124]
Impala	*Aepyceros melampus*	[98]
Lesser hedgehog tenrec	*Echinops telfairi*	[99]
Lowland gorilla	*Gorilla gorilla gorilla*	[125]
Mink	*Mustela vison*	[126]
Mouse	*Mus musculus*	[66, 127]
Nine-banded armadillo	*Dasypus novemcinctus*	[90]
Okapi	*Okapia johnstoni*	[121]
Red deer	*Cervus elaphus*	[118]
Sheep	*Ovis aries*	[116]
Spotted hyena	*Crocuta crocuta*	[99]
Springbok	*Antidorcas marsupialis*	[118]
Tammar wallaby	*Macropus eugenii*	[59]
Water buffalo	*Bubalus bubalis*	[128]
White-lipped peccary	*Tayassu pecari*	[119]

### Glycosylation and the development of the great obstetrical syndromes

The Great Obstetrical Syndromes are of the utmost concern for the practicing obstetrician [107]. The existing data indicate that these syndromes are due to defects in deep implantation. Perhaps one of the most puzzling of these syndromes is preeclampsia (PE). The thoughtful obstetrician Jeffcoate referred to PE as “the Disease of Theories” because of the numerous research challenges associated with this pathological condition [108]. This disorder can be broadly divided into two classes, sometimes referred to as maternal and placental, though in some cases a mixture of the two types is observed [109, 110]. Placental PE is the result of poor placentation during early pregnancy. PE has been hypothesized to be the result of: (i) a disruption of vascular remodeling leading to hypoxia; and/or (ii) an aberrant immune response directed against the allogeneic fetus [111]. There is strong evidence indicating that both processes play crucial roles in the clinical manifestation of PE.

Sgambati and coworkers previously analyzed the distribution of sugar residues in human placentas from uncomplicated pregnancies and those affected by different hypertensive disorders (pregnancy-induced hypertension (PIH), PE, PE with hemolysis, elevated liver enzymes and low platelets (HELLP) syndrome) [112]. They employed ConA, WGA, PNA, SBA, DBA, UEA, GNA, DSA, MAA and DSA in combination with other chemical and enzymatic treatments to perform this analysis. They reported a 40 % increase in ConA binding to STB and CTB in placentas derived from patients that developed PE or PE with HELLP syndrome compared to TB derived from women that developed pregnancy-induced hypertension or that delivered without complications. No binding sites for DBA or SBA were detected on STB and CTB in the placentas of patients with uncomplicated deliveries. However, substantial binding of these lectins was observed on STB and CTB associated with the placentas of patients that developed PIH, PE or PE with HELLP syndrome. Binding sites for SNA were expressed on STB from the placentas of patients that developed PE with HELLP, but not on TB from any other patient group analyzed in this study [112]. Clearly, these results indicate that a shift in glycosylation is occurring during the development of these obstetrical syndromes, but how these changes impact this condition remains to be defined.

Potential shifts in glycosylation have recently been indicated during the development of preterm birth. Integrin β1 was isolated from villous samples obtained 6–9 weeks of gestation from placentas obtained from early spontaneous miscarriage and normal controls [113]. Binding of L-PHA, a lectin that specifically recognizes N-glycans bearing the Galβ1-4GlcNAcβ1-6 Man sequence was decreased in integrin samples isolated from patients that experienced a miscarriage compared to controls. By contrast, the level of binding of E-PHA to integrin β1 substantially increased in samples derived from miscarriage patients compared to normal pregnancies. These shifts in glycosylation were correlated with the level of the N-acetylglucosaminyltransferase enzymes known as Mgat 5 and GnT-III that add β1-6 linked and the bisecting GlcNAc to the trimmanosyl core of N-glycans, respectively [113]. Whether these shifts in glycosylation play a role in the development of preterm birth remains to be defined.

## Conclusions

Many studies have focused considerable effort on defining glycosylation in the mammalian uterus and placenta. Nonetheless, the functional roles of glycans have not been explicitly defined. Studies focused in this area could be extremely valuable in the human, where the cause of many of the Great Obstetrical Syndromes remain enigmatic [107]. Though powerful methods of both genetic and epigenetic analysis are currently available to analyze uterine and trophoblast function, no definitive cause of these major obstetrical syndromes has been determined. Evidence is now available indicating that the glycosylation of STB and CTB are undergoing substantial changes during the development of hypertensive disorders of pregnancy including PIH, PE and PE with HELLP syndrome [112]. Shifts in the glycosylation of β1 integrin have been detected in the placentas of women who developed preterm birth [113]. The time has now come to employ biochemical and ultrasensitive MS tool to analyze the pathways for glycosylation in the uterus in both the pregnant and nonpregnant women during both normal and aberrant physiological states. The possibility that aberrant glycosylation could play a major role in the development of the Great Obstetrical Syndromes should now be seriously considered.

## Abbreviations

GdA: Amniotic glycoforms of glycodelin
GdF: Follicular fluid glycoforms of glycodelin
GdS: Seminal plasma glycoforms of glycodelin
HELLP: Hemolysis, elevated liver enzymes and low platelets
MS: Mass spectrometric. A list of the standard abbreviations for lectins referred to in text is provided in Table 1
